# Annotations

**Published:** 1909-06-05

**Authors:** 


### ANNOTATIONS.

### Medicine at Oxford University.

The very active sympathy which the Chancellor of Oxford University, Lord Curzon, displays towards the medical school of that seat of learning is both gratifying to the medical profession and a most noteworthy proof of the statesmanlike breadth of view which the late Viceroy of India is now confidently trusted to exhibit towards important questions of public policy. Only a score or two of years ago an Oxford Chancellor whose studies had been classical would have as soon been expected to concern himself with the advancement of the scientific and medical faculties as to attend the dinner of the Oxford Graduates' Medical Club; yet Lord Curzon has done both, and his speech to the members of the latter shows how keenly alive he is to the necessities of the former. Fifty years ago, we gather, the Oxford Medical School practically had no existence; to-day it supplies sixty-eight members of the teaching staffs of hospitals to which medical schools are attached or of Universities. Among the schemes of internal reformation which are engaging the most serious consideration of the authorities of the University, the encouragement and extension of the medical school are very prominent. No longer can it be said with the least shadow of justification what Lord Curzon said of Oxford generally in the middle of the nineteenth century, that the condition of science there might almost be compared with that of the Dark Ages, and that the attitude towards medical science in particular and science in general was one of suspicion if not of active hostility. In 1850 there was not a single scientific laboratory in Oxford: bearing this fact in mind, and the Chancellor's speech as well, it may be confidently hoped that the wonderful progress made since then is only a foretaste of that which will be accomplished before fifty-nine more years have rolled away.

### The Spirit Duty.

Since the first introduction of the Budget the effect of the increase of the proposed duty of an extra three shillings and ninepence per gallon of spirit upon the price of chloroform, tinctures, and other pharmaceutical preparations has been widely discussed. The result of the various expressions of opinion by physicians, pharmacists, and other interested persons was that a decidedly benevolent neutrality was evinced by the Chancellor of the Exchequer towards the exemption of spirit destined for such commercial purposes from the operation of the increase. As The Hospital went to press last week the probability of concession along these lines seemed particularly hopeful, but the text of the Finance Bill, as finally introduced, does not seem to contain any confirmation of these hopes. It has already been pointed out in these columns (May 8, p. 147) that chloroform and ether are neither the luxury of the idle rich nor the vice of the intemperate poor, but a necessity; and it now appears that the price of these drugs (or rather of those varieties of them made from dutiable spirit) will be raised at least tenpence a pound. Such an impost on an article which is to all intents a necessity of life, and already pays about 250 per cent. duty *ad valorem*, is indefensible from any point of view—humanitarian, utilitarian, common-sense, or any other. It is notable that at an inquest held on May 21 on the body of a child which died at St. Bartholomew's Hospital, the Coroner elicited the fact that this extra duty would cost the hospital from £400 to £500 a year extra on the drug bills alone. Such a statement is one which should of itself suffice to convince the Chancellor—or, failing him, the House of Commons—of the absolutely immoral nature of the proposed taxation. We cannot believe that Mr. George will prove obdurate over this matter if it is properly pressed upon his attention; and it ought to be the especial business of the medical men in the House to secure some definite concession on so important a matter with the least possible delay.

### The Medical Council's Finances.

In our news columns last week we published an Abstract of the Presidential Address, delivered at the opening of the eighty-ninth session of the General Medical Council by Sir Donald Macalister; but, although several matters of general interest and importance were dealt with, we refrained from commenting upon them because they had already received the fullest consideration in recent numbers of The Hospital. Whilst the General Minutes afford little excuse for comment or criticism, the Report of the Finance Committee is full of material for thought. This year the Committee has to announce a deficit of more than £650, which comes as a shock after three years of large balances on the pleasant side of the ledger. The reasons for a heavy deficit are stated to be beyond the Council's control, and a study of the accounts tends to confirm this assertion. In the matter of receipts the three branches of the Council received £290 less from registration fees, original and additional, than in 1907, and of this £259 was due to the falling-off in Scotland. But it is in the expenses of the General Council that the principal cause of the adverse balance is to be found—here the fees were larger by £656, law expenses by £302, and miscellaneous expenses by £142. This was because the Council sat for thirteen days in 1908 as against eight days in 1907, and the extra length was entirely due to two penal cases. The sessions cost about £200 per diem in fees and hotel expenses; and these two cases, for the protraction of which the Council disclaims all responsibility, cost the Council £2,100. In the words of the Finance Committee's Report, the Council "must give a fair hearing to a practitioner charged with so grave an offence as 'infamous conduct in a professional respect'; but, when the case is unduly protracted, the financial results are disastrous." But for these two cases, instead of a deficit of £650 there would have been a surplus of £1,100. The Report does not emphasise the word "unduly"; but it will no doubt strike many of our readers that there lies the crux of the matter. Such an appalling waste of public (and professional) money must surely not be allowed to occur again.

